# Glycerin supplementation strategies for three or seven days affects oxidative stress, follicle dynamics and ovulatory response in Morada Nova sheep

**DOI:** 10.1590/1984-3143-AR2020-0025

**Published:** 2022-06-01

**Authors:** Mariza Araújo Morais de Moura Andrade, Juliana Paula Martins Alves, Iolly Tábata Oliveira Marques Galvão, Camila Muniz Cavalcanti, Maria Raquel Lopes Silva, Alfredo José Herrera Conde, Alessandra Façanha Bezerra, César Carneiro Linhares Fernandes, Dárcio Italo Alves Teixeira, Davide Rondina

**Affiliations:** 1 Faculdade de Medicina Veterinária, Universidade Estadual do Ceará, Fortaleza, CE, Brasil

**Keywords:** energy diet, short-term supplementation, glutathione peroxidase, ovulation rate

## Abstract

This study examined the effect of glycerin supply strategies in different short-term protocols on follicular dynamics and ovulatory rate in Morada Nova sheep. Eighteen Morada Nova ewes with body condition > 2.9 had their estrus and follicular waves synchronized using three injections of prostaglandin analogue at seven-day intervals. All animals received the same diet during 21 days, which consisted of a total mixed ration (TMR) based on chopped elephant grass and concentrate twice daily. In the control group (n=9), ewes were fed the TMR diet. In the other four groups, ewes received 150 mL of glycerol daily, supplied as an oral drench or mixed in the TMR during three or seven days prior to the application of the third PGF2 alfa analogue. These groups were named as follows: Drench3d (n=10), Drench7d (n=8), TMR3d (n=9) and TMR7d (n=9). Follicle dynamics were monitored by ultrasonography, and plasma glucose and glutathione peroxidase levels were measured at the third prostaglandin administration. Six days after the final PGF2 alfa analogue dose, ovulatory rate was measured by laparoscopy. Glucose was higher (P< 0.001) in the glycerin-treated groups than in control group (83.7 ± 1.7 vs. 68.4 ± 4.5 mg. dL^-1^; P < 0.001). Ewes in the TMR3d, Drench7d and TMR7d groups had a greater (P < 0.001) number of large follicles (≥ 3 < 5 mm), and the presence of follicles larger than 5 mm was observed. In the same groups, at the third PGF2 alfa analogue dose, a greater (P < 0.001) number of growing follicles (> 3 mm) and a larger size of the largest follicle (P < 0.001) were also recorded. Ovulation rate was 30% higher in the groups that received glycerin for seven days (1.6 ± 0.1 53 vs. 1.1 ± 0.1; P < 0.05), and they also exhibited a 38% reduction in glutathione peroxidase. Thus, the use of glycerin in Morada Nova sheep as a source of energy in short-term supplementation for increase ovulation rate is an efficient strategy when provided for seven days, either orally or in the feed.

## Introduction

Brazil is the second largest biodiesel produce, thus, glycerin emerges as a low cost alternative energy source for ruminant feeding, contributing to minimizing the costs of intensive production (Ornaghi et al., 2016). One of the current strategies for making use of glycogenic precursors in small ruminants is short-term supplementation, which is aimed at optimizing folliculogenesis. In sheep, glucose infusion for three or five days is able to trigger an “acute” metabolic signaling, which is shown to be advantageous in stimulating the functionality of the ovary, extending the final stages of folliculogenesis and consequently increasing ovulation rate (Dupont and Scaramuzzi, 2016). The use of glycerol as a short-term supplement would help solving two problems: the utilization of this by product and the low reproductive performance of the flock.

Glycerol administered in the form of glycerin to ruminants promotes metabolic changes-by increasing plasma glucose and insulin concentrations. The animal response is largely dependent on the dose used and the form of administration. While intravenous glycerol infusion (170 mL), promotes blood glucose and insulin peaks 1-2 h after administration in sheep, an oral drench (270-300 mL) extends the peaks from 6 to 12 h after administration in sheep and goats (Rodrigues et al. 2015; Ferraro et al. 2016; Kalyesubula et al., 2019). Moreover, excessive glycemic stimulation can lead to the occurrences of hyperglycemia, reducing oocyte and embryo quality (van Eetvelde et al., 2017). This is because the associated increase in insulin levels induces epigenetic changes; a decrease in the glutathione enzyme and, possibly increasing oxidative stress; resulting in an accumulation of reactive oxygen species (ROS) (Uhde et al., 2018).

Previous studies have demonstrated the effectiveness of glycerin supplementation (six days) in follicle stimulation used as an oral drench in sheep (Oliveira et al., 2016) and in improving embryo quality in goats (Silva et al., 2014). Despite the positive results described in the literature, some aspects still limit its use in extensive systems, since the best form of administration of this energy supplement is still debatable. The use of glycerin in the form of oral drench provides ease of application and allows the control of the dose administered (Porcu et al., 2018), but it requires frequent handling of the animals. In contrast, the administration of glycerin in the feed favors the application of continuous treatments (Van Cleef et al., 2018).

Nonetheless, it does not allow for an efficient control of the effective dose ingested, especially if animals are feed collectively.

We hypothesized that the oral drench of glycerin would be more effective than the administration in the feed, and that there would be no differences between 3 and 7 days supplementation on the number of follicles stimulated to ovulate. Also, that the oral drench would have no effect on glutathione enzyme activity, and indicative of oxidative stress.

Therefore, the present study proposes to determine whether the addition of glycerin in oral form or included in the feed for three or seven days influences glucose concentrations, oxidative stress, follicle dynamics and ovulatory response in Morada Nova ewes.

## Methods

### Animals and experimental design

The experiment was conducted at the Agricultural Experimental Farm Dr. Esaú Accioly de Vasconcelos, in the municipality of Guaiúba, Ceará State, located at 4º2 23”S and 38º38’14”W, from March 2019 to January 2020. This area, characterized by a constant photoperiod regimen, has a warm, tropical, sub-humid climate with a mean annual rainfall and temperature of 904.5 mm and 26-28º C, respectively.

**Figure 1:**
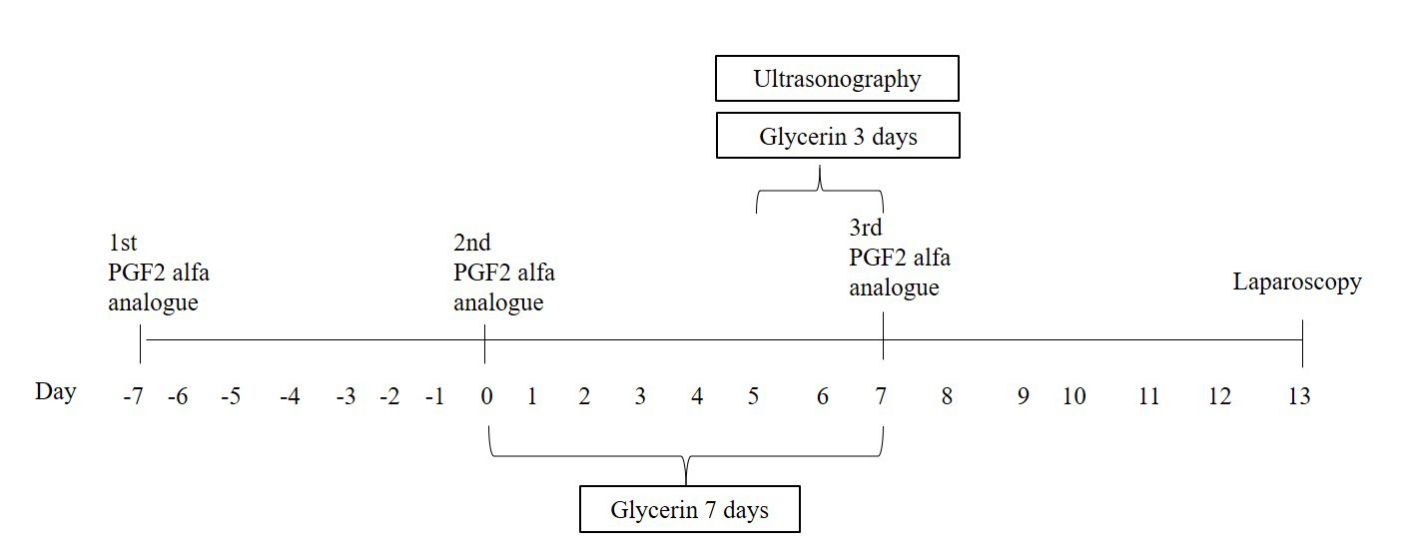
Time line of hormonal protocol, ultrasound assessment and laparoscopy during glycerin administration for three or seven days.

All procedures performed in this study were approved by the Ethics Committee on Animal Experimentation of UECE (approval no. 3450166/2014, CEUA-UECE). Eighteen animals were used in three trials. In each trial, the groups to be tested were randomly chosen. For each group, a similar number of animals was maintained (n = 9). In addition, a minimum 90-day interval between trials was maintained to allow sufficient time between treatments applied in each trial. The handling conditions and food level remained similar during the experimental trials. Forty-five multiparous Morada Nova ewes were distributed in a randomized experimental design with five group treatments. The Morada Nova breed does not show reproductive seasonality. Sheep body mass index was calculated as follows: BMI = ((body weight (kg)/ withers height (m)/body length (m))/10. The animals were homogenous (P > 0.05) in terms of body mass index (8.1 ± 0.8; overall mean ± SD), age (2.3 ± 0.9 years) and body condition score > 2.9 (scale of 1 to 5). Their body condition score was equivalent to that of an animal with subcutaneous fat thickness at the loin of 3.1 ± 0.4 mm and loin depth mean measured in the lumbar region of 16.8 ± 2.5 mm. Overall means for body weight, withers height and body length at the start of the trials were 28.1 ± 3.3 kg, 62.9 ± 2.6 cm and 62.7 ± 1.9 cm, respectively (P > 0.05). Ewes received the same diet, which consisted of a total mixed ration (TMR) based on chopped elephant grass and concentrate (ground grain corn, 60%; wheat bran, 25%; soybean meal, 10%; and mineral and vitamin mixture, 5%). The TMR was prepared in a water solution and supplied to meet the nutritional requirements of adult sheep (NRC, 2007). The experimental animals were kept in collective stalls, where they had free access to mineral supplement and water. The diets were provided twice daily (07:00 and 15:00 h) and refusals were collected daily and weighed weekly to determine the intake of the dietary supplements by the animals. All ewes had estrus and follicle waves synchronized as described by Viñoles et al., (2010), by administering three injections of 100 μg of the prostaglandin analogue (PGF2 alfa analogue) D-Cloprostenol (Prolise^®^ - Tecnopec, São Paulo, Brazil), at seven days intervals (Figure 1). The third application of PGF2 alfa analogue was aimed at promoting estrus and ovulation simultaneously in all ewes.

The ewes received energy supplementation based on 150 mL glycerol in the 138 form of glycerin solution (containing 99% glycerin and water at a 9:1 ratio). All animals 139 were supplemented daily for three or seven days before the third application of PGF2 140 alfa analogue (Figure 1). In two groups, glycerin solution was administrated to the ewes as an oral drench in water (9:1), 1 h after feeding, for three days (Drench3d group; 142 n=10) or seven days (Drench7d group; n=8) prior to the third PGF2 alfa analogue injection. In the two other groups, glycerin was supplemented in the TMR for three (TMR3d group; n=9) or seven days (TMR7d group; n=9) prior to the third PGF2 alfa analogue injection. Glycerin was added daily, per ewe, in the TMR diet during the supplementation period. The TMR diet was kept as the control group (n=9), and ewes received a drench of 150 mL saline solution for 7 days. The estimated energy value of glycerol was 0.77 Mcal of ME (Mach et al., 2009). Based on these values, glycerin supplementation increased the ME density of the diet by 47% daily, in relation to the TMR provided in the control treatment.

### Ultrasound assessments and laparoscopy

Ovarian images were obtained with a B-mode ultrasound equipment using a 5- MHz linear probe (model DP-2200Vet, Shenzhen Mindray Bio-Medical Eletronics Co., Ltd., China) that was inserted into a plastic handle so that the probe could be manipulated externally in the rectum. Examinations were also recorded on videotape (one cassette per ewe), followed by image capture and analysis using Image J software (Image J, National Institutes of Health, Millersville, USA), which was previously calibrated. In the hormonal protocol, day 0 was defined as the first application of PGF2 alfa analogue. Ultrasonography was performed twice daily from Day 5 to the last injection of PGF2 alfa analogue (Figure 1). An ovarian follicle wave was defined as the emergence of a group of small follicles (< 3 mm in diameter) that originated one or more large follicles (>5 mm). An intermediate diameter (≥3 mm to <5 mm) was considered a medium-size follicle.

Subcutaneous fat thickness at the loin (SFTL) and loin depth (LD) were measured at beginning and end of the experiment before laparoscopy, using the same ultrasound device with a linear 5-MHz transducer. The ewes were held still and the transducer was placed linearly in the region between the 3rd and 4th lumbar vertebrae (Silva et al., 2014). To measure the structures of interest, ultrasonographic examinations were recorded in the form of videos, followed by the capture and measurement of images for each structure using Image J software (Image J, National Institutes of Health, Millersville, USA), which was previously calibrated.

Six days after the last injection of PGF2 alfa analogue (Figure 1), ovulatory response was quantified by laparoscopy, through a morphological classification of corpus luteum as described before (Guido et al., 2003).

### Blood sampling, glucose and glutathione peroxidase assays

Blood samples were taken at the third prostaglandin injection by jugular venipuncture, using heparinized vacutainer tubes (Labor import, Wei Hai, 179 China), prior to the morning feeding. The samples were centrifuged at 600 *g* for 15 min, and the obtained plasma was stored at −20 °C for subsequent quantification of the metabolites. Plasma glucose concentrations were determined using an automated biochemical analyzer (Mindray BS 120, Mindray®) with commercial kits (Bioclin®, 183 Quibasa — Minas Gerais, Brazil). The sensitivity of the assay kit was 1.31 mg/dL for glucose. Glutathione peroxidase (GPx) was analyzed using a semi-automated biochemical analyzer (Randox RX Monza TM, Randox Laboratories®, Crumlin, UK) and commercial kits (Randox Laboratories®, Crumlin, UK) with 75 U/L sensitivity for GPx.

### Statistical analysis

Data were subjected to analysis of variance (ANOVA) using a general linear model procedures (Statistica v. 13.4.0.14, TIBCO Software, Inc., Palo Alto, CA, USA). The statistical model considered the fixed effect of the group-diet (Control, Drench3d, TMR3d, Drench7d and TMR7d), time (interval of assessments used during the time length of supplementation) and group × time interaction, for the measurements of body weight, SFTL, LD and follicle-dynamics. For plasma metabolites and ovulatory response, the factor used was the group-diet of the supplementation treatment. Pairwise comparisons were performed by the Newman-Keuls test. Metabolite concentrations, number of follicles as obtained by ultrasonography and ovulatory response data were log- transformed (logx10).

## Results

Table 1 shows the results of the *in vivo* performance of the experimental groups. The Drench7d and TMR7d groups were heavier than the control group at the end of the respective tests. Loin depth did not differ as a function of the diet, whereas the thickness of adipose tissue exhibited a statistical difference between the control and TMR7d treatment groups (data not shown). There was an effect of glycerin administration for plasma glucose concentration (Table 1). All groups treated with glycerin showed higher blood glucose values as compared with the control group. There were no differences between groups in relation to food intake.

**Table 1:** Means and standard errors of body weight, loin depth, subcutaneous fat thickness, glucose and follicular dynamics in Morada Nova ewes treated for three or seven days with glycerin as oral drench or included in total mixed ration.

**Parameter**	**N**	**Group**					**P-value**		
		**Control**	**Drench3d**	**TMR3d**	**Drench7d**	**TMR7d**	**Group**	**Time**	**G × T**
In vivo performance*									
Body weight, kg		28.6 ± 0.9ac	28.3 ± 1.2a	31.2 ± 1.0cd	33.1 ± 1.0bd	33.6 ± 1.1bd	< 0.001	0.95	0.47
LD, mm**		17.7 ± 0.6	16.9 ± 0.9	16.6 ± 0.4	17.8 ± 0.5	17.2 ± 0.7	0.43	0.35	0.33
SFTL, mm***		3.5 ± 0.1a	3.4 ± 0.2ab	3.4 ± 0.2ab	3.1 ± 0.1ab	2.9 ± 0.1b	0.01	0.27	0.47
Metabolites*									
Glucose, mg.dL^-1^		68.4 ± 4.5a	87.3 ± 4.2b	89.0 ± 3.2b	80.9 ± 1.7b	76.8 ± 2.0b	< 0.001	-	-
*Follicular dynamics*									
Follicles < 3mm, n	675	1.8 ± 0.2a	2.4 ± 0.2be	1.3 ± 0.1c	1.9 ± 0.1ab	2.3 ± 0.1de	< 0.001	< 0.001	0.09
Follicles ≥ 3 < 5 mm, n	279	0.4 ± 0.1a	0.2 ± 0.1b	1.2 ± 0.1c	0.8 ± 0.1d	0.8 ± 0.1d	< 0.001	0.20	0.05
Follicles > 5 mm, n	13	-	-	0.03 ± 0.02	0.07 ± 0.03	0.04 ± 0.02	0.29	0.96	0.67
Mean follicle size, mm	967	2.5 ± 0.1a	2.1 ± 0.1b	3.1 ± 0.1c	2.9 ± 0.1d	2.6 ± 0.05ad	< 0.001	< 0.001	0.56

* Measured at the third application of PGF2 alfa analogue; **Loin depth; ***Subcutaneous fat thickness at the loin.

**Figure 2:**
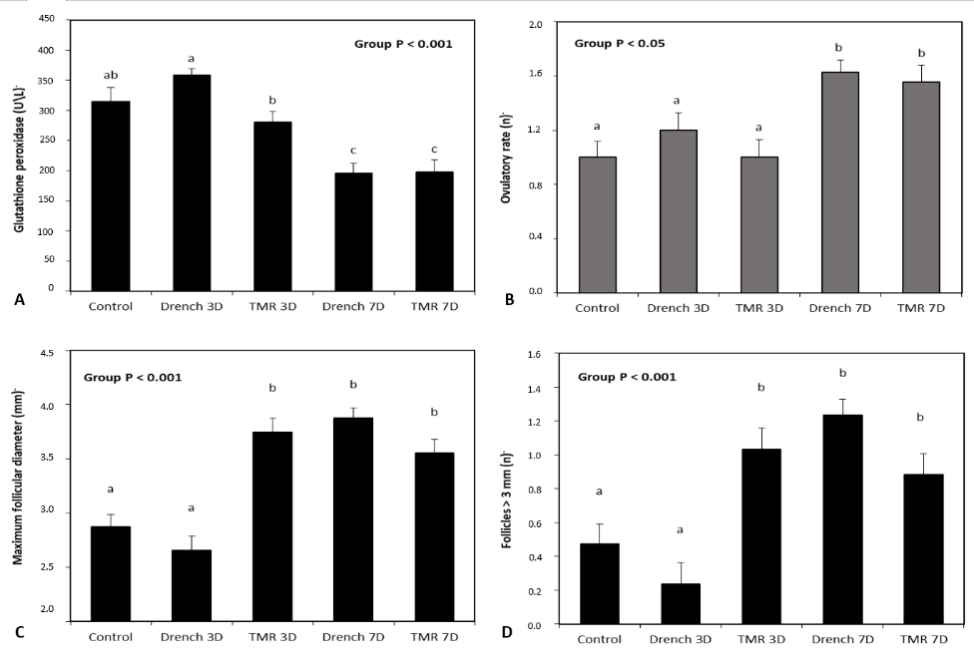
Means and standard errors of plasma glutathione peroxidase concentration (A), maximum follicular diameter (B), number of follicles > 3 mm (C) and ovulatory rate (D). The data shown in Figures A, B and C were measured at the third application of PGF2 alfa analogue. Ovulatory rate was determined by laparoscopy six days after the third application of PGF2 alfa analogue. Analysis of variance results for the effect of group is represented in the figure. Values in **Figure 2B** and 2C are shown as the mean of follicles counted by ultrasonography at the third application of PGF2 alfa analogue.

In all treatments, there was a reduction in the number of follicles <3 mm (Table 1) during the administration of glycerin (time effect P <0.001). On average, the TMR3d group had the lowest number of follicles of this class. The TMR3d, Drench7d and TMR7d groups showed a higher number of follicles between 3 and 5 mm when compared with the other treatment groups. No statistical significance was observed for the effects tested in the follicle class with a diameter greater than 5 mm. These types of follicle structures were reported in the TMR3d, Drench7d and TMR7d groups (Table 1). Average follicle diameter increased during the measurement interval in all treatments (time effect P <0.001). The largest mean diameters were found in the TMR3d and Drench7d groups. Figure 2 shows the results related to the plasma glutathione peroxidase rate and the follicle dynamics parameters observed after the glycerin administration period. The same figure also shows the ovulation rate (Figure 2D) determined six days after the third injection of PGF2 alfa analogue. The Drench7d and TMR7d treatment groups exhibited the lowest concentrations of glutathione peroxidase (Figure 2A) and an average increase of 33% in ovulation rate when compared with the other groups (Figure 2D).

Mean while, the TMR3d, Drench7d and TMR7d groups had the largest follicle diameters (Figure 2B) and a higher number of follicles > 3 mm (Figure 2C).

## Discussion

The hypothesis that the oral drench of glycerin would be more effective that the administration in the feed, and that there would be no differences between 3 and 7 days supplementation on the number of follicles stimulated to ovulate, was not accepted. Supplementing Morada Nova ewes with glycerin was successful when provided for seven days, regardless of whether glycerin was applied as a drench or via feed. Moreover, the three- day glycerin inclusion in TMR provided better follicle dynamics than the oral drench. The addition of glycerin in the feed increased the number of growing follicles and increased-follicle size. The inclusion of glycerin in the TMR for three days allowed the appearance of large follicles with a diameter greater than 5 mm, similarly to what occurred in the two treatments involving a longer period. The second part of our hypothesis was also rejected, since 7 days glycerin supplementation, regardless of the administration form, revealed a marked reduction in oxidative stress and an increase in ovulation rate.

The lack of a clear change in the subcutaneous muscle and fat masses between the start and end of the experiment was expected, given the age and nutritional status of the animals and the short period of supplementation. In adult animals with high body condition, fat deposition is mainly visceral, rather than subcutaneous or muscular. In sheep as well as other ruminants in the maturity stage, the diet does not change lean mass, considering that the growth rate in these animals reaches a maturity point at which the deposition of adipose tissue exceeds that of other tissues (Irshad et al., 2013). Viñoles et al. (2005) using a 6 days supplementation treatment, found a similar body condition score but an increase in body weight in the supplemented group associated to the difference in the amount of feed supplied to the experimental groups. In our study, all groups received the same TMR diet plus glycerin, which explains the lack of differences in body weight.

The increase of plasma glucose concentrations found in the present study was probably due to the high rate of glycerol absorption when glycerin was administered as a drench or TMR. This finding corroborates previous studies with Morada Nova ewes supplemented via drench for six days (Oliveira et al., 2016) and goats supplemented with glycerin in TMR for seven days (Alves et al., 2019). In goats, Silva et al. (2014) supplying 100 mL or 200 mL of glycerol, administered as a drench, found an increase in plasma insulin and glucose levels in animals treated with 200 mL. In addition to glucose, increases in insulin and leptin concentrations are also reported in the literature from short- term nutritional supplementation, and relatively higher concentrations thereof are associated with a higher ovulation rate (Viñoles et al., 2010).

In the present study, the glycerin supplementation for seven days promoted a marked increase in the ovulation rate, which reached an average 270 value of 1.6. In goats, Rodrigues et al. (2015) reported ovulation rates of 1.15 in control group and 0.89 in the 300 ml glycerin treated group. We do not have a clear explanation for these contradictory results, but species differences in the response to glycerin supplementation, the dose administered and the synchronization protocol used may be part of the answer. However, in Morada ewes, glycerin supplementation for 7 days increased ovulation rate to levels reported using 400 IU equine chorionic gonadotropin (Souza Rodrigues et al., 2004).

The 7 days supplemented groups exhibited the lowest concentrations of glutathione peroxidase associated to an increase in ovulation rate, but the opposite occurred in the 3 days supplemented groups. The differential response between 3 vs 7 days may be associated to the stage of the follicle wave when the supplementation started. While in 7 days groups it started before the expected time of emergence of the first wave of the cycle and lasted during the occurrence of the wave, in 3 days groups it started around the time of follicle selection (Viñoles et al., 2005, 2010). The exposure of follicles to increased glucose-insulin levels at different times of their development, may have promoted epigenetic changes at cell level (Uhde et al., 2018). This may have resulted in differential production of reactive oxygen species (ROS), including superoxide (O2–) and non-reactive H2O2 (Turrens, 2003), that are involved in the onset of apoptosis in the antral follicles (Ciani et al., 2015). We suggest that supplementing glycerin by the time of follicle selection in the groups treated for 3 days, may explain the increase in plasma glutathione peroxidase and the reduction in ovulation rate observed.

## Conclusion

We conclude that under the experimental conditions of the present study, the administration of 150 mL glycerol daily, for seven days, in the form of glycerin solution as an oral drench or in the feed, represents a promising strategy of energy supplementation for Morada Nova ewes. The application of glycerin has been shown to induce, also in animals with high nutritional status, an efficient signaling in the control of oxidative stress and in ovarian response, leading to gains greater than 30% in ovulation rate.

Nevertheless, our results did not reveal reproductive advantages regarding the form of supply—oral or in the feed. For these reasons, the authors suggest that the definition of the best glycerin application strategy should be carefully analyzed by the producer, considering also other factors such as the costs of applying the treatment in relation to the type of management used at the farm.
